# Tetrahedral framework nucleic acids ameliorate cholestatic liver disease by activating Wnt/β-catenin signaling and promoting ERK1/2 phosphorylation

**DOI:** 10.1093/rb/rbaf017

**Published:** 2025-03-20

**Authors:** Jiaming Zhou, Chenxi Tang, Xin Song, Yating Wang, Bingru Lin, Mengchi Lin, Zixin Xu, Shihua Lin, Chengfu Xu, Chaohui Yu

**Affiliations:** Department of Gastroenterology, Zhejiang Provincial Clinical Research Center for Digestive Diseases, the First Affiliated Hospital, Zhejiang University School of Medicine, Hangzhou 310003, China; Department of Gastroenterology, Zhejiang Provincial Clinical Research Center for Digestive Diseases, the First Affiliated Hospital, Zhejiang University School of Medicine, Hangzhou 310003, China; Department of Gastroenterology, Zhejiang Provincial Clinical Research Center for Digestive Diseases, the First Affiliated Hospital, Zhejiang University School of Medicine, Hangzhou 310003, China; Department of Gastroenterology, Zhejiang Provincial Clinical Research Center for Digestive Diseases, the First Affiliated Hospital, Zhejiang University School of Medicine, Hangzhou 310003, China; Department of Gastroenterology, Zhejiang Provincial Clinical Research Center for Digestive Diseases, the First Affiliated Hospital, Zhejiang University School of Medicine, Hangzhou 310003, China; Department of Gastroenterology, Zhejiang Provincial Clinical Research Center for Digestive Diseases, the First Affiliated Hospital, Zhejiang University School of Medicine, Hangzhou 310003, China; Department of Gastroenterology, Zhejiang Provincial Clinical Research Center for Digestive Diseases, the First Affiliated Hospital, Zhejiang University School of Medicine, Hangzhou 310003, China; Department of Gastroenterology, Zhejiang Provincial Clinical Research Center for Digestive Diseases, the First Affiliated Hospital, Zhejiang University School of Medicine, Hangzhou 310003, China; Department of Gastroenterology, Zhejiang Provincial Clinical Research Center for Digestive Diseases, the First Affiliated Hospital, Zhejiang University School of Medicine, Hangzhou 310003, China; Department of Gastroenterology, Zhejiang Provincial Clinical Research Center for Digestive Diseases, the First Affiliated Hospital, Zhejiang University School of Medicine, Hangzhou 310003, China

**Keywords:** cholestatic liver disease (CLD), tetrahedral framework nucleic acids (TFNAs), Wnt/β-catenin signaling pathway, ERK1/2 phosphorylation, α-naphthyl isothiocyanate (ANIT)

## Abstract

Cholestatic liver disease (CLD) is characterized by disruptions in bile formation, secretion and excretion, leading to progressive liver injury, inflammation and fibrosis. Effective treatments to halt or reverse the progression of CLD remain limited. The Wnt/β-catenin signaling pathway has been implicated in the regulation of bile acid homeostasis and liver regeneration, playing a complex role in CLD pathophysiology. Tetrahedral framework nucleic acids (TFNAs), a class of anti-inflammatory and antioxidant DNA nanomaterials, have shown potential in promoting mammalian cell proliferation through activation of cell cycle and proliferation-related signaling pathways. However, their therapeutic potential in CLD has not been fully explored. In this study, we investigated the effects of TFNAs in an α-naphthyl isothiocyanate (ANIT)-induced mouse model of CLD. TFNAs demonstrated the ability to enter hepatocytes, where they activated the Wnt/β-catenin signaling pathway and enhanced ERK1/2 phosphorylation. These molecular changes resulted in significant improvements in liver injury markers, bile acid metabolism and liver regeneration. Complementary *in vitro* experiments revealed that TFNAs reduced hepatocyte apoptosis and oxidative stress, while promoting cell viability and proliferation. Histological analysis confirmed that TFNAs treatment mitigated liver necrosis, reduced ductular reactions and decreased neutrophil infiltration, highlighting their anti-inflammatory and tissue-protective effects. These findings provide compelling evidence that TFNAs can ameliorate CLD by modulating key signaling pathways involved in hepatocyte survival, regeneration and bile acid homeostasis. Collectively, our findings highlight the therapeutic potential of TFNAs as a novel treatment for CLD and paves the way for further exploration of nanomaterials in liver disease therapy.

## Graphical abstract

**Figure:**
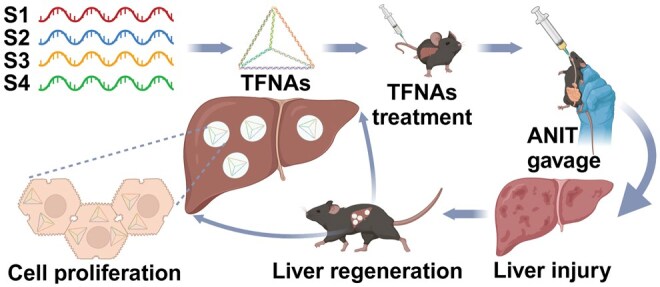

## Introduction

Cholestatic liver diseases (CLDs) are characterized by impaired hepatobiliary secretion of bile acids (BAs), leading to their accumulation both intrahepatically and extrahepatically [1]. Chronic forms of CLD, particularly primary biliary cholangitis (PBC) and primary sclerosing cholangitis (PSC), often progress to liver fibrosis and cirrhosis, ultimately causing liver failure, which can be fatal [2]. Current medical therapy for PBC is effective in only about half of the patients, and no effective therapy exists for PSC [3]. For those with end-stage liver disease, liver transplantation remains the sole effective option, although some PSC patients may experience relapse even after transplantation [2].

One of the therapeutic strategies for CLD involves reducing BAs synthesis or inhibiting their uptake into hepatocytes [4]. A key pathway potentially involved in the reduction of BAs synthesis is the Wnt signaling pathway. β-Catenin, the primary downstream effector of Wnt signaling, acts as a second messenger by binding to T cell factor/lymphoid enhancer factor (TCF/LEF) transcription factors, thereby promoting the transcription of downstream genes [5]. Emerging evidence indicates that the Wnt/β-catenin signaling pathway plays a role in almost all aspects of liver biology. The initial discovery came from studies showing that liver-specific β-catenin knockout (KO) mice exhibited defects in BA and cholesterol homeostasis [6]. Further research revealed that KO mice fed a cholic acid diet displayed elevated hepatic and serum BAs levels, bile ductular reactions, increased pericellular fibrosis and dilated, misshapen bile canaliculi [7]. Interestingly, while some studies found that the loss or inhibition of β-catenin could reduce total BAs and alleviate hepatic injury by expediting farnesoid X receptor (FXR) activation following bile duct ligation (BDL) or a 3,5-diethoxycarbonyl-1,4-dihydrocollidine (DDC) diet [8]. Other evidence suggested that β-catenin may prevent the progression of CLD in the Mdr2 knockout murine model [9]. These findings imply that the Wnt/β-catenin signaling pathway may play a complex and multifaceted role in the development of CLD.

In recent years, the use of DNA to construct multidimensional materials has gained considerable attention, largely due to the pioneering work of Seeman [10]. Among various DNA-based polyhedrons, tetrahedral DNA nanostructures have garnered the most interest due to their unparalleled programmability, functional diversity, structural stability, natural biocompatibility, biodegradability and ability to be internalized by cells [11, 12]. Tetrahedral framework nucleic acids (TFNAs), formed by four specific single-strand DNAs (ssDNAs) [13], have emerged as a potential therapeutic strategy for several diseases, due to their anti-inflammatory and antioxidant properties [14–16]. Notably, recent studies have shown that TFNAs can promote the proliferation of mouse AML12 hepatocytes, mouse L929 fibroblasts and human periodontal ligament stem cells by activating the Wnt/β-catenin signaling pathway [17–19].

This study aims to investigate whether TFNAs can ameliorate CLD and whether this effect is associated with the activation of the Wnt/β-catenin signaling pathway, as well as anti-inflammatory and antioxidant mechanisms. To test this hypothesis, we used mouse AML12 hepatocytes and primary hepatocytes (PHCs) for *in vitro* experiments, and an α-naphthyl isothiocyanate (ANIT)-induced CLD model for *in vivo* experiments. Collectively, our findings demonstrate that TFNAs ameliorate ANIT-induced CLD through activation of the Wnt/β-catenin and ERK1/2 signaling pathways, along with anti-inflammatory and antioxidant effects.

## Materials and methods

### Synthesis and characterization of the TFNAs

Four specially designed ssDNAs (Supplementary Table S1) were synthesized and purified to prepare a 10 μM stock solution (Sangon Biotech, Shanghai, China). Equal concentrations of these ssDNAs and TM buffer (pH 8.0, containing Tris-HCl and MgCl_2_) were mixed in a microtube and gently vortexed. The mixture was then self-assembled into TFNAs through one-pot annealing (95°C for 10 min, followed by 4°C for 20 min) using a PCR machine.

To confirm successful TFNAs assembly, 2% agarose (BS081, Biosharp) gel mixed with GelRed (BS354, Biosharp) was used for electrophoretic identification. The morphology of the TFNAs was determined by atomic force microscopy (AFM, Bruker Dimension Icon, Germany), and their structure and size were further verified by transmission electron microscopy (TEM, HITACHI H-7650, Japan).

### Experimental animals

All animal experiments were conducted in strict accordance with the guidelines for the Care and Use of Laboratory Animals provided by the National Institutes of Health. The study protocol was approved by the Institutional Animal Care and Use Committee of The First Affiliated Hospital, Zhejiang University School of Medicine (Approval Number: 2024-1632).

Male C57BL/6 mice (7 weeks old; Ziyuan Laboratory Animal Technology Co., Ltd, Hangzhou, China) were acclimated for one week to the experimental environment. For the ANIT-induced cholestasis model, mice were randomly assigned to three groups: the negative control group (NC group, Ctrl + Olive oil), the ANIT control group (ANIT group, Ctrl + ANIT), the ANIT TFNAs treatment group (TFNAs group, TFNAs + ANIT). Each group received a tail vein injection prior to ANIT gavage. The TFNAs group was injected with TFNAs at a concentration of 250 nM, while the NC and ANIT groups were injected with a control solvent. Following the injections, ANIT gavage was administered to the ANIT and TFNAs groups, while the NC group received olive oil gavage. After 48 h of gavage and overnight fasting, the mice were euthanized, and blood samples were immediately collected for biochemical analysis. Liver tissue was harvested, weighed, perfused with PBS to remove blood and subsequently cut into small pieces, frozen in dry ice and stored at −80°C for later analysis. Liver tissue samples were also fixed in 4% paraformaldehyde for paraffin embedding.

### Isolation and culture of mouse primary hepatocytes

Mouse PHCs were isolated from 8-week-old male C57BL/6 mice using collagenase digestion, as previously described [20]. After attachment, hepatocytes were treated with different concentrations of TFNAs for 12 h, followed by stimulation with 50 μM ANIT for 24 h. Cell lysates were then collected for quantitative real-time polymerase chain reaction (PCR) and western blot analysis [20].

### Cellular uptake of the TFNAs

To assess cellular uptake, TFNAs were self-assembled using cyanine-5 (Cy5)-labeled ssDNAs, and their permeability was evaluated via confocal microscopy [13, 21]. Mouse AML12 hepatocytes were incubated with Cy5-TFNAs for varying durations (0, 2, 4, 8 and 12 h) after stepwise starvation. After incubation, the cells were washed with PBS in a dark environment and fixed with 4% paraformaldehyde. Cytoskeletons were stained with fluorescein isothiocyanate (FITC)-phalloidin (Solarbio, Beijing, China), and nuclei were labeled with 2-(4-amidinophenyl)-6-indolecarbamidine dihydrochloride (DAPI; Solarbio, Beijing, China).

### Cell viability assay

AML12 hepatocytes and mouse PHCs were seeded into 96-well culture plates at a density of 8000–10 000 cells per well. Cells were randomized into seven groups: negative control group, TFNAs (0 nM) + ANIT, TFNAs (62.5 nM) + ANIT, TFNAs (125 nM) + ANIT, TFNAs (250 nM) + ANIT, TFNAs (375 nM) + ANIT, TFNAs (500 nM) + ANIT. After incubation with TFNAs at various concentrations (0, 62.5, 125, 250, 375 and 500 nM) for 12 h, the cells were exposed to 50 μM ANIT for 24 h to establish an *in vitro* model of CLD. Cell viability was assessed using the cell count kit-8 (CCK8) assay.

A calcein acetoxymethyl ester/propidium iodide (Calcein/PI) live/dead viability assay kit (Beyotime, Shanghai, China) was used to further evaluate cell viability. Live cells stained with Calcein emitted green fluorescence, while dead cells stained with PI emitted red fluorescence. The ratio of live to dead cells was determined through Calcein and PI double staining.

### Quantitative real-time PCR

As we reported previously [20], total RNA was isolated from mouse liver tissue or cell lysates using TRIzol reagent (AG21101, Accurate Biotechnology) according to the manufacturer’s instructions. RNA was reverse transcribed into cDNA, and quantitative real-time PCR was performed. Gene expression levels were normalized to GAPDH, used as an internal control, and calculated using the 2^−ΔΔCT^ method. Primer sequences are listed in Supplementary Table S2.

### Western blot analysis

Total proteins were extracted from mouse liver tissues and cells using RIPA buffer (FD008, FUDE) with protease (FD1001, FUDE) and phosphatase (FD1002, FUDE) inhibitors, as described previously [20]. Protein concentrations were adjusted to 2 μg/μl using a BCA kit (P0011, Beyotime). Samples (20 μg per lane) were separated on 8% or 10% SDS-PAGE gels and transferred onto polyvinylidene difluoride membranes (0.22 μm, Millipore). Membranes were blocked with 5% bovine serum albumin (BSA) in Tris-buffered saline Tween (TBST, G0004, Servicebio) for 1 h at room temperature, followed by overnight incubation with primary antibodies. After washing, membranes were incubated with HRP-conjugated secondary antibodies (1:5000, BL051A/BL052A, Biosharp) for 1 h at room temperature. Protein bands were visualized using an enhanced chemiluminescence kit (FD8020, FUDE). Primary antibodies are listed in Supplementary Table S3.

### Liver histological, immunofluorescence and immunohistochemical staining analysis

As previously described [20, 22], paraffin sections of the liver were subjected to hematoxylin and eosin (H&E) staining, immunohistochemistry (IHC) and immunofluorescence (IF) staining. AML12 hepatocytes were stained for β-catenin via IF staining. Images were captured using a confocal laser scanning microscope (FV-3000, Olympus, Japan) and a confocal microscope platform (STELLARIS 5, Leica, Germany). Sources and dilutions of antibodies for IHC and IF are listed in Supplementary Table S4.

### Statistical analysis

Statistical analyses were performed using GraphPad Prism (version 9.5). All experiments were conducted at least three times, and data are presented as mean ± standard deviation. Independent samples *t*-tests or Mann–Whitney *U*-tests were used for comparisons between two groups. Multiple group comparisons were performed using one-way ANOVA or the Kruskal–Wallis test. A *P*-values < 0.05 was considered statistically significant.

## Results

### Characterization and endocytosis of the TFNAs

**Figure 1.**
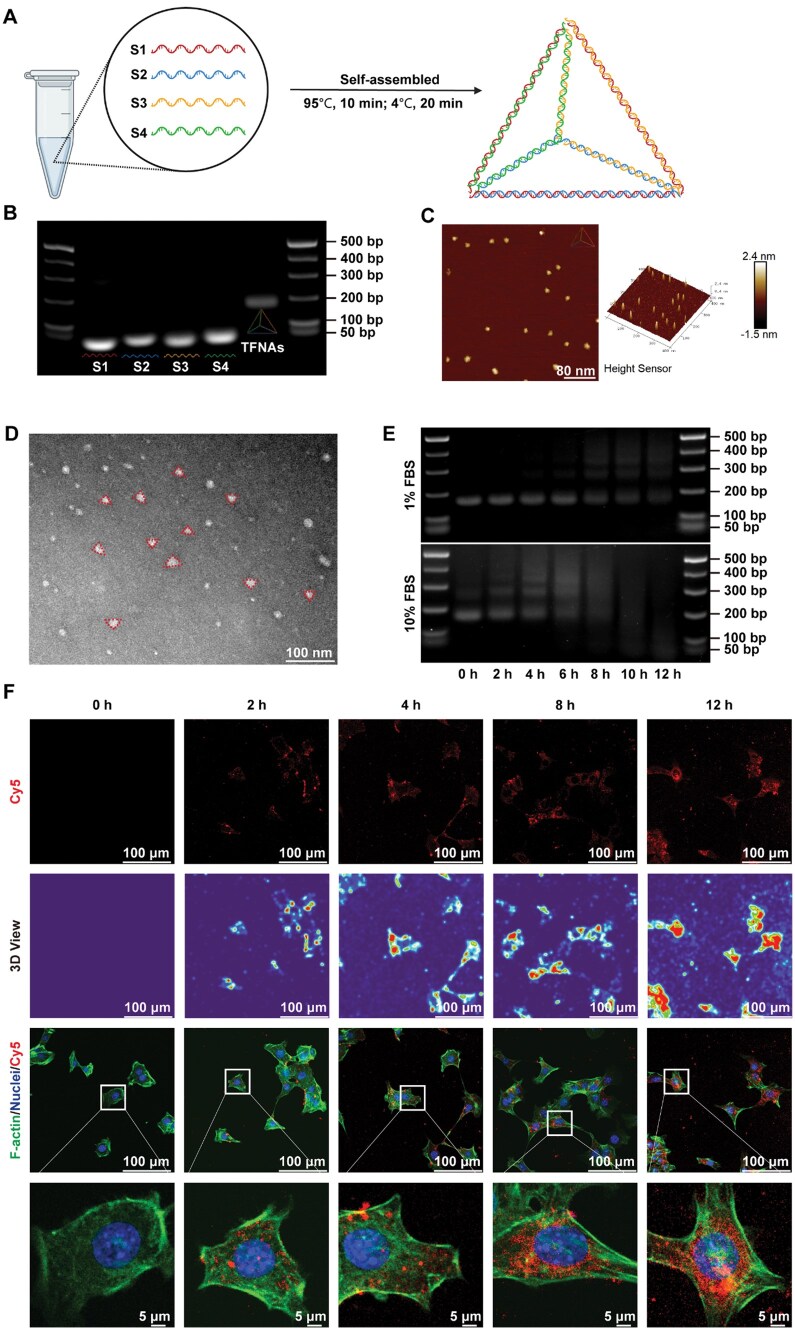
Synthesis, characterization, and endocytosis of the TFNAs. (**A**) Schematic diagram of TFNAs formation. (**B**) Identification of the successful synthesis of TFNAs using AGE. (**C**) Surface morphology of the TFNAs observed by atomic force microscopy. (**D**) Structure of the TFNAs observed by transmission electron microscopy. (**E**) Stability evaluation of TFNAs in FBS using AGE. (**F**) Cellular uptake of TFNAs detected by confocal microscopy (red: TFNAs; green: cytoskeleton; blue: nuclei). AGE, agarose gel electrophoresis; TFNAs, tetrahedral framework nucleic acids.

Four specifically designed ssDNAs were self-assembled into TFNAs using a one-pot annealing method in a PCR machine (Figure 1A) [13, 21]. The successful assembly of the TFNAs was verified using 2% agarose gel electrophoresis (AGE) (Figure 1B), where the size of TFNAs was approximately 200 base pairs (bp), corresponding to the expected total size of the four ssDNAs. Atomic force microscopy (AFM) and transmission electron microscopy (TEM) confirmed that the TFNAs form triangular structures with sizes ranging from 10 to 20 nm (Figure 1C and D).

The stability of TFNAs in a biological environment was evaluated by incubating them in fetal bovine serum (FBS) at different time intervals. AGE indicated that 250 nM TFNAs maintained structural stability in 1% FBS-containing medium for up to 6 h. However, degradation commenced between 6 and 12 h of incubation, with a progressive decline in structurally intact TFNAs observed during this period, although residual intact structures remained detectable at the 12 h time point (Figure 1E and Supplementary Figure S1A). These findings informed our decision to conduct a stepwise serum starvation regimen under *in vitro* experimental conditions, with TFNAs applied in a 1% FBS-supplemented medium throughout the experimental protocol. To examine cellular uptake, TFNAs labeled with cyanine dye (Cy5-TFNAs) were incubated with AML12 hepatocytes and PHCs. Over time, the red fluorescent signals from Cy5 increased in intensity, remaining stable in the cytoplasm for 12 h (Figure 1F). Furthermore, we conducted comprehensive biodistribution analysis using an *in vivo* imaging system (IVIS) to track the spatiotemporal dynamics of Cy5-TFNAs in C57BL/6 mice (Supplementary Figure S1B). After intravenous injection into animals, the fluorescent signal of Cy5-TFNAs increases rapidly in the liver within the first 15 min. Subsequently, the kidneys begin to accumulate a fluorescent signal after 30 min and gradually increase. This time-dependent biodistribution demonstrates TFNAs' rapid hepatotropic targeting followed by renal clearance mechanisms. H&E staining of major organ demonstrates that TFNAs administration at therapeutic concentrations does not induce detectable toxicity or histopathological alterations in murine models (Supplementary Figure S1C).

### TFNAs alleviate hepatocyte injury after ANIT treatment *in vitro*

**Figure 2.**
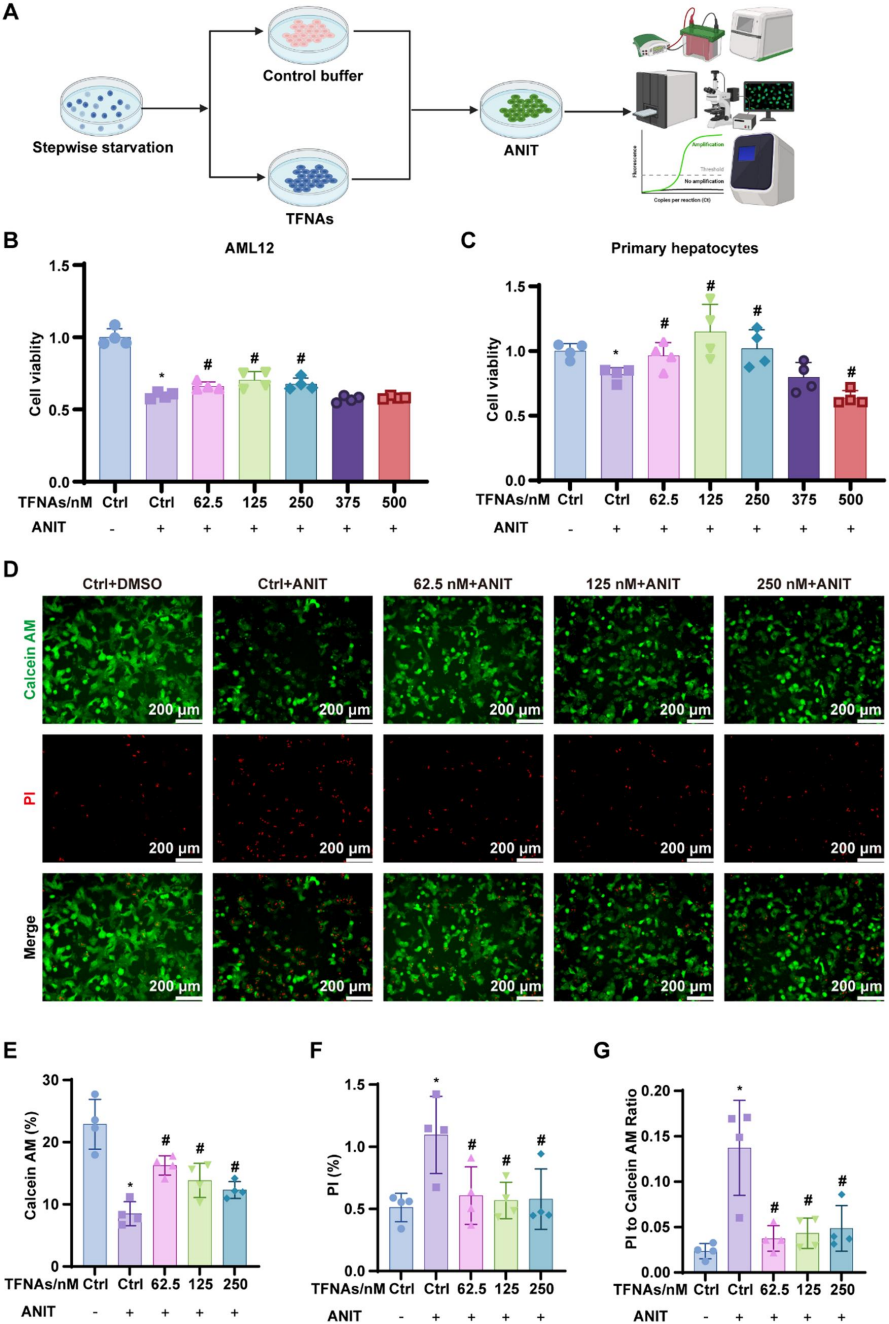
TFNAs alleviate hepatocyte injury in ANIT-induced CLD model *in vitro*. (**A**) Schematic of *in vitro* experiments with cell viability, RT-qPCR, WB and IF analysis. (**B–C**) CCK8 assay of AML12 hepatocytes and PHCs (*n *=* *4). (**D–G**) Calcein/PI live/dead viability assay of PHCs (*n *=* *4). The data are presented as the mean ± SD. *P <* *0.05 vs. the negative control group; ^#^*P *<* *0.05 vs. the ANIT-treated control group. ANIT, α-naphthyl isothiocyanate; Calcein, calcein acetoxymethyl ester; CCK8, cell count kit-8; CLD, cholestatic liver disease; PHCs, primary hepatocytes; PI, propidium iodide; RT-qPCR, quantitative real-time polymerase chain reaction; TFNAs, tetrahedral framework nucleic acids; WB, western blot.

AML12 hepatocytes and PHCs were exposed to TFNAs at different concentrations (0, 62.5, 125, 250, 375 and 500 nM) for 12 h, followed by ANIT treatment to induce liver injury (Figure 2A). Prior to initiating *in vitro* investigations, we systematically validated the intracellular persistence of TFNAs under ANIT-induced pathological conditions. Fluorescence microscopy analysis of Cy5-TFNAs in AML12 hepatocytes demonstrated sustained intracellular retention throughout the 24 h ANIT exposure (Supplementary Figure S2A). The CCK8 assay revealed that TFNAs, particularly at 125 nM, significantly improved cell viability compared to the untreated ANIT group (Figure 2B and C). To further assess cell viability and cytotoxicity, Calcein AM and PI staining were employed. TFNAs-treated PHCs had more viable cells and fewer dead cells after ANIT treatment compared to the untreated ANIT group (Figure 2D). The results demonstrated a higher ratio of live (Calcein AM-stained) to dead (PI-stained) cells in the TFNAs-treated groups (Figure 2E–G), suggesting that TFNAs effectively prevented ANIT-induced hepatocyte injury. Similar protective effects were observed in AML12 hepatocytes (Supplementary Figure S2B–E).

### TFNAs activate Wnt/β-catenin and ERK1/2 pathways to alleviate hepatocyte injury

**Figure 3.**
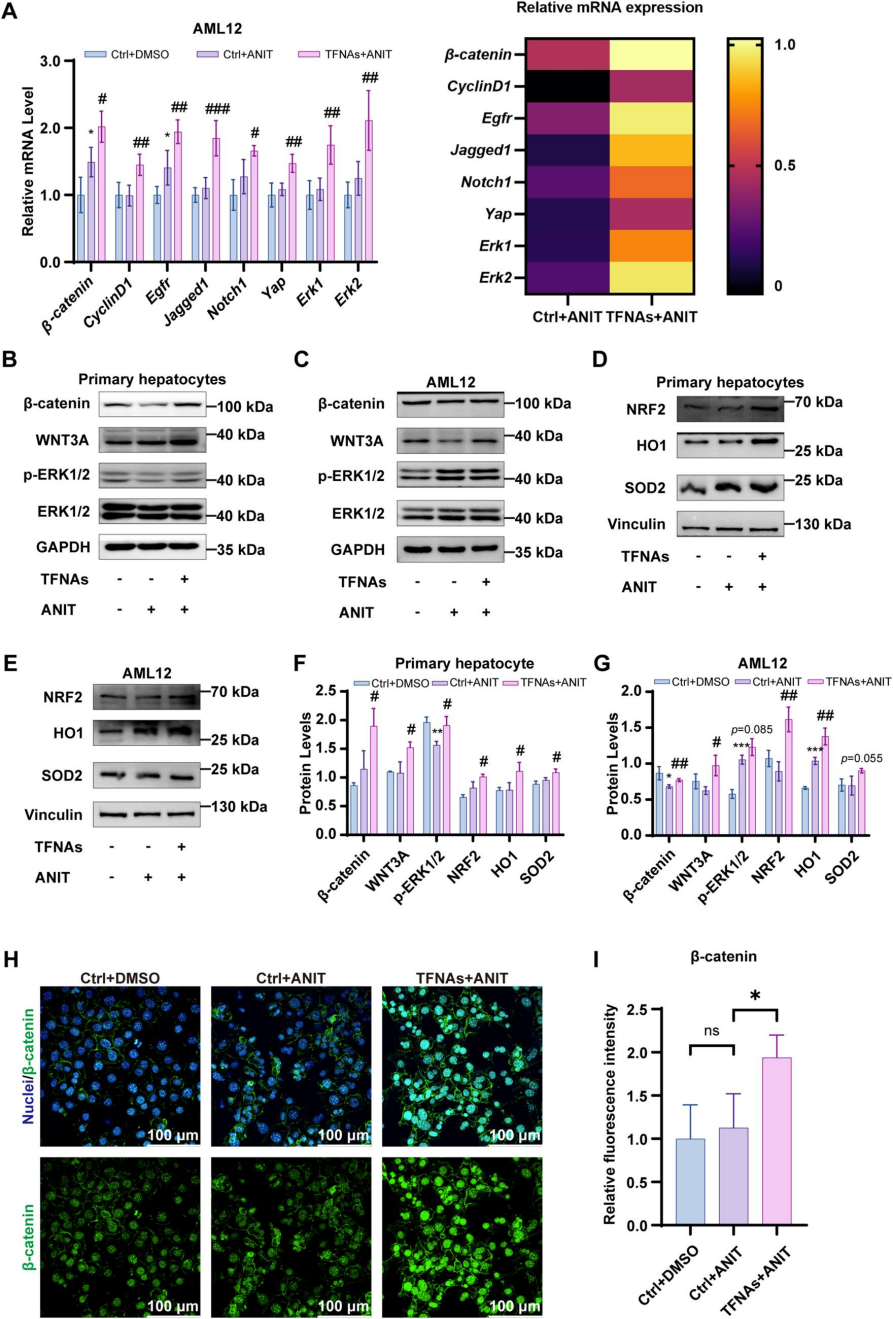
TFNAs activate Wnt/β-catenin and ERK1/2 pathways to alleviate hepatocyte injury in ANIT model *in vitro*. (**A**) The mRNA levels of *β-catenin*, *CyclinD1*, *Egfr*, *Jagged1*, *Notch1*, *Yap* and *Erk1/2* were analyzed by RT-qPCR and quantitative evaluation (*n *=* *4). (**B–E**) The protein levels of β-catenin, WNT3A, p-ERK1/2, ERK1/2, NRF2, HO1 and SOD2 were detected by WB. (**F–G**) Quantitative evaluation of WB results (*n *= 3). (**H–I**) IF confocal microscope of β-catenin (blue: nuclei; green: β-catenin) (*n* = 3). The data are presented as the mean ± SD. *P < 0.05, **P <* *0.01, ***P <* *0.001 vs. the negative control group; ^#^*P *<* *0.05, ^##^*P *<* *0.01, ^###^*P *<* *0.001 vs. the ANIT-treated control group. ANIT, α-naphthyl isothiocyanate; RT-qPCR, quantitative real-time polymerase chain reaction; TFNAs, tetrahedral framework nucleic acids; WB, western blot.

To investigate the mechanisms by which TFNAs protect against ANIT-induced hepatocyte injury, several signaling pathways related to cell proliferation were analyzed. mRNA expressions of *Wnt*, *Notch* and *EGFR* signaling pathway components were significantly upregulated in TFNAs-treated AML12 hepatocytes compared to the untreated group (Figure 3A). The heat map provides a more visual representation of the above mRNA with higher expression levels in the TFNAs-treated group compared with the untreated ANIT group. At the same time, Western blot analysis confirmed increased protein levels of WNT3A, β-catenin and phosphorylated ERK1/2 in the TFNAs-treated group (Figure 3B and C). Additionally, TFNAs promoted the expression of antioxidant proteins NRF2, HO1 and SOD2 (Figure 3D–G). Immunofluorescence staining further confirmed increased β-catenin nuclear localization in hepatocytes treated with TFNAs (Figure 3H). These findings suggest that TFNAs protect hepatocytes by activating the Wnt/β-catenin and ERK1/2 pathways, reducing hepatocyte injury in the ANIT-induced model *in vitro*.

### TFNAs ameliorate ANIT-induced CLD *in vivo*

**Figure 4.**
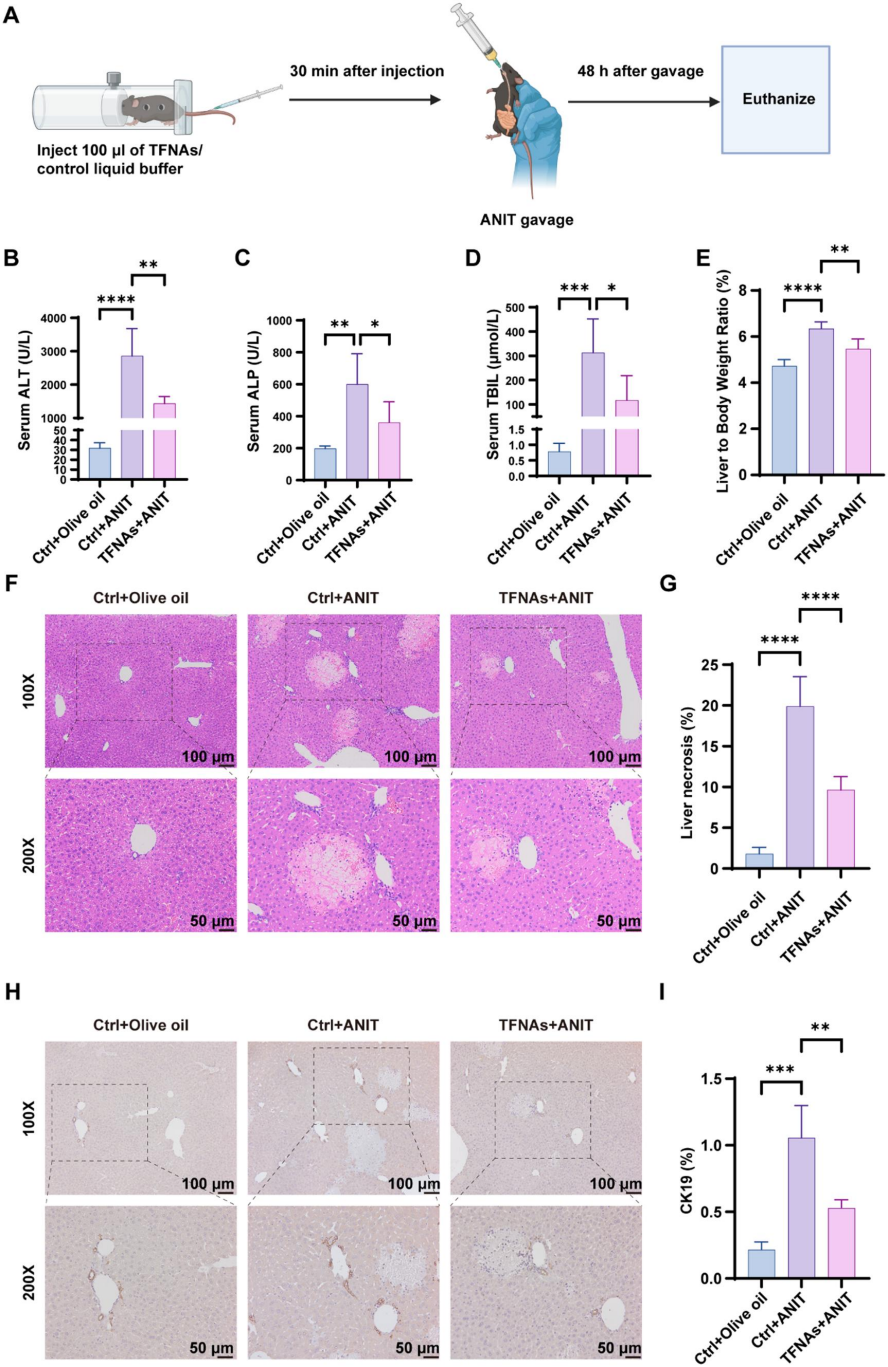
TFNAs ameliorate ANIT-induced CLD *in vivo*. (**A**) Schematic of the *in vivo* animal experiment design. (**B–D**) Serum ALT, ALP and TBIL levels in ANIT model (*n *=* *5). (**E**) Liver-to-body weight ratio of mice after ANIT gavage (*n *=* *5). (**F**) H&E staining of liver sections (*n *=* *5). (**G**) Quantitative evaluation of liver necrosis area (*n *=* *5). (**H**) IHC staining of CK19 in liver sections (*n *=* *5). (**I**) Quantitative evaluation of CK19-positive area (*n *=* *5). The data are presented as the mean ± SD. *P <* *0.05, **P <* *0.01, ***P <* *0.001, ****P <* *0.0001. ALP, alkaline phosphatase; ALT, alanine transaminase; ANIT, α-naphthyl isothiocyanate; CK19, cytokeratin 19; CLD, cholestatic liver disease; H&E, hematoxylin and eosin; IHC, immunohistochemistry; TBIL, total bilirubin; TFNAs, tetrahedral framework nucleic acids.

The therapeutic potential of TFNAs was further evaluated in an ANIT-induced mouse model of CLD (Figure 4A). Serum biochemical markers such as alanine transaminase (ALT), alkaline phosphatase (ALP) and total bilirubin (TBIL) were dramatically elevated in the ANIT group compared to the NC group, while TFNAs treatment reduced these markers (Figure 4B–D). The liver-to-body weight ratio, which reflects liver injury, was also lower in the TFNAs group (Figure 4E). Histological analysis via H&E staining revealed that liver necrosis was significantly reduced in the TFNAs group compared to the ANIT group (Figure 4F and G). Immunohistochemical staining of CK19, a marker for ductular reaction, showed a reduction in CK19-positive cells in TFNAs-treated livers, further indicating amelioration of liver injury (Figure 4G and H).

### TFNAs promote liver regeneration and reduce inflammation thorough activation of the Wnt/β-catenin, ERK1/2 and NRF2 signaling pathway in ANIT-induced CLD

**Figure 5.**
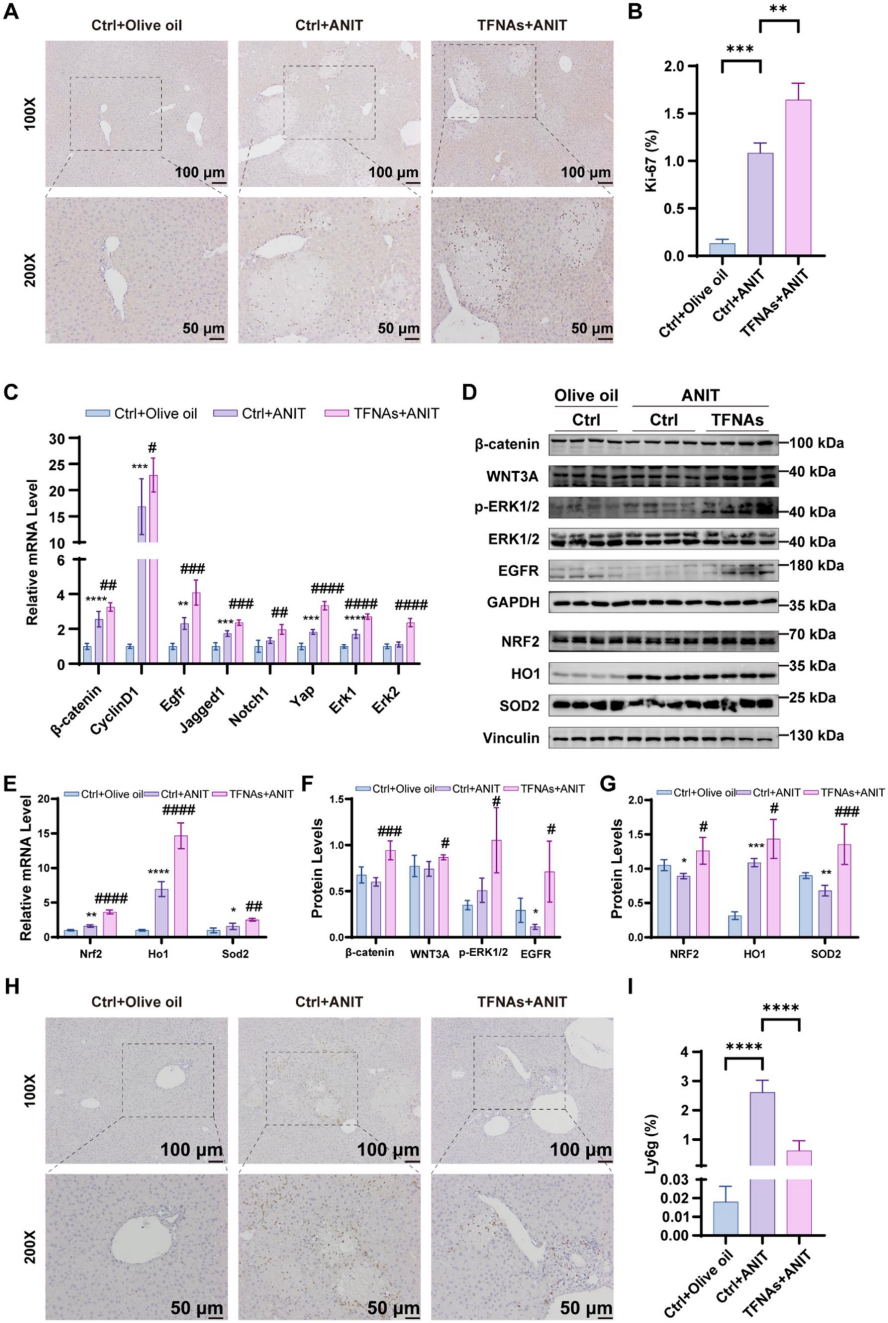
TFNAs promote liver regeneration and reduce inflammation in ANIT-induced CLD. (**A**) IHC staining of Ki-67 in liver sections (*n *=* *5). (**B**) Quantitative evaluation of Ki-67-positive area (*n *=* *5). (**C**) The mRNA level of *β-catenin*, *CyclinD1*, *Egfr*, *Jagged1*, *Notch1*, *Yap* and *Erk1/2* was analyzed by RT-qPCR and quantitative evaluation (*n *=* *5). (**D**) The protein levels of β-catenin, WNT3A, p-ERK1/2, ERK1/2, EGFR, NRF2, HO1 and SOD2 were detected by WB. (**E**) The mRNA level of *Nrf2, HO1* and *Sod2* was analyzed by RT-qPCR and quantitative evaluation (*n *=* *5). (**F–G**) Quantitative evaluation of WB results (*n *=* *5). (**H**) IHC staining of Ly6g in liver sections (*n *=* *5). (**I**) Quantitative evaluation of Ly6g-positive area (*n *=* *5). The data are presented as the mean ± SD. *P <* *0.05, **P <* *0.01, ***P <* *0.001, ****P <* *0.0001 vs. the negative control group; ^#^*P *<* *0.05, ^##^*P *<* *0.01, ^###^*P *<* *0.001, ^####^*P *<* *0.0001 vs. the ANIT-treated control group. ANIT, α-naphthyl isothiocyanate; CLD, cholestatic liver disease; RT-qPCR, quantitative real-time polymerase chain reaction; TFNAs, tetrahedral framework nucleic acids; WB, western blot.

Immunohistochemical analysis of Ki-67, a proliferation marker, revealed increased hepatocyte proliferation in TFNAs-treated mice compared to the ANIT group (Figure 5A and B). In order to explore the underlying mechanism of TFNAs in promoting liver regeneration, mRNA expressions of *Wnt*, *Notch* and *EGFR* signaling pathway were analyzed. The *β-catenin*, *CyclinD1*, *Egfr*, *Notch1*, *Jagged1*, *Yap* and *Erk1/2* mRNA expression levels were upregulated in the TFNAs group compared with the ANIT group (Figure 5C). Similarly, western blot analysis showed elevated levels of WNT3A, β-catenin and phosphorylated ERK1/2 in the TFNAs-treated livers (Figure 5D and F). The expression of antioxidant proteins NRF2, HO1 and SOD2 was also upregulated in the TFNAs-treated mice (Figure 5D and G), consistent with *in vitro* findings. Moreover, mRNA levels of *Nrf2*, *Ho1* and *Sod2* were also elevated in the TFNAs group (Figure 5E). Elevated BAs in CLD hepatocytes recruit neutrophils via chemokine signaling, promoting hepatic inflammation [23]. Therefore, we examined the infiltration of neutrophils by immunohistochemical staining with Ly6g, a specific marker for neutrophiles. TFNAs treatment reduced neutrophil infiltration, as indicated by Ly6g-positive staining, suggesting reduced hepatic inflammation (Figure 5H–I). However, no significant differences in macrophage infiltration (F4/80 staining) were observed between the ANIT and TFNAs groups (Supplementary Figure S3A–B). Similar results were observed with immunofluorescence analysis (Supplementary Figure S3C). Collectively, TFNAs enhance liver regeneration via Wnt/β-catenin and ERK1/2 pathways activation, while reducing inflammation through NRF2-mediated antioxidant responses, thereby ameliorating ANIT-induced CLD *in vivo*.

## Discussion

In this study, we explored the therapeutic potential and regulatory mechanisms of an innovative framework nucleic nanomaterial, known as TFNAs, in addressing hepatocytes injury during CLD. Our key findings demonstrate that: (i) TFNAs ameliorate ANIT-induced hepatocyte injury *in vitro* (Figure 2); (ii) TFNAs play a protective role in the ANIT-induced CLD model, mitigating liver injury and restoring BA homeostasis (Figure 4); and (iii) TFNAs activate the Wnt/β-catenin signaling pathway and promote ERK1/2 phosphorylation, providing antioxidant effects both *in vivo* and *in vitro* (Figures 3 and 5). These results suggest that TFNAs hold promise as therapeutic agents for cholestasis, potentially modulating liver inflammation and injury through these pathways.

Hepatic lobules are characteristic structures of the liver that can be subdivided into three regions: zone 1 consists of hepatocytes in the area of the portal triad; zone 3 consists of hepatocytes near the central vein; hepatocytes between these two regions make up zone 2 [24]. The liver consists of zonated regions with distinct metabolic functions, including BA synthesis in pericentral hepatocytes (zone 3) [5]. Studies have shown that the Wnt/β-catenin signaling pathway is a key regulator of liver zonation, with β-catenin particularly active in pericentral hepatocytes [25, 26]. BA synthesis enzymes like cholesterol 7α-hydroxylase (CYP7A1) and sterol 27-hydroxylase (CYP27) are expressed in pericentral hepatocytes, implying that they may be regulated by the Wnt/β-catenin signaling pathway [27]. Recent studies have shown that liver-specific β-catenin knockout mice exhibit BA accumulation, and worsen intrahepatic cholestasis and liver fibrosis in a 0.5% cholic acid-supplemented diet [6, 7]. Similarly, disruption of β-catenin plays an aggravating role in the Mdr2 knockout murine model of CLD, highlighting its importance in liver protection [9]. However, in other CLD models such as BDL and DDC diet, β-catenin inhibition may reduce BAs and alleviate liver injury, indicating context-dependent effects [8]. Collectively, these findings imply that the Wnt/β-catenin signaling pathway may play a complex and multifaceted role in the development of CLD.

It has shown that TFNAs enhance cell proliferation of mouse L929 fibroblasts via activating the Wnt/β-catenin pathway [17]. Chen *et al.* also found that TFNAs promoted hepatocyte proliferation by activating the Wnt/β-catenin signaling pathway, thereby alleviating liver injury and enhancing liver regeneration in 70% partial hepatectomy, acetaminophen overdose and carbon tetrachloride-exposed mice [18]. It is reasonable to speculate that TFNAs may modulate cholestasis by activating the Wnt/β-catenin signaling pathway. In our study, we observed that TFNAs upregulate β-catenin in AML12 hepatocytes and mouse PHCs *in vitro*, leading to amelioration of ANIT-induced liver injury (Figures 2 and 3). *In vivo*, TFNAs improved BA homeostasis and attenuated inflammation in the ANIT model, reinforcing the protective role of Wnt/β-catenin in CLD (Figures 4 and 5). These findings align with previous research that highlights Wnt/β-catenin as a potential target in CLD.

Additionally, TFNAs promoted the expression of EGFR (Figure 5), which is known to protect against cholestatic injury and regulate BA synthesis [28]. Recently, it has been reported that the promotion of corneal epithelial wound healing by TFNAs may be related to the upregulation of ERK1/2 phosphorylation level [29]. It is also shown that phosphorylation of ERK1/2 plays a critical role in cholangiocyte repair during biliary damage [30]. Given that ANIT is metabolized by hepatocytes and secreted into bile to damage cholangiocytes [31], our observation of TFNAs-induced ERK1/2 phosphorylation in the liver suggests that TFNAs may alleviate cholestasis by enhancing cholangiocyte repair via ERK1/2 activation (Figure 5). However, we observed that TFNAs promoted ERK1/2 phosphorylation in hepatocytes *in vitro*. In the future, further research is needed to determine whether TFNAs specifically promote ERK1/2 phosphorylation in hepatic progenitor cells for cholangiocyte repair.

The Wnt signaling pathway plays a dual role in CLD, with its effects varying based on disease stage, cell type and intercellular signaling interactions. As a key regulator of cell proliferation, Wnt signaling influences the cell cycle at multiple checkpoints, promoting hepatocyte proliferation and facilitating tissue repair and functional restoration in the liver [32, 33]. Additionally, during cholestatic liver injury, Wnt activation can induce hepatocyte reprogramming toward a biliary phenotype, contributing to bile duct regeneration [34]. The pathway also plays a crucial role in bile duct epithelial repair by stimulating cholangiocyte proliferation and differentiation [35, 36]. However, Wnt signaling is not universally beneficial. β-Catenin, a central effector of the Wnt pathway, can suppress FXR activation in hepatocytes, disrupting the regulation of CYP7A1, a key enzyme in BA synthesis, thereby exacerbating BA accumulation and cholestatic injury [8]. Furthermore, Wnt/β-catenin signaling is implicated in hepatic stellate cell activation, with studies demonstrating that inhibition of this pathway mitigates liver fibrosis [37, 38]. Additionally, β-catenin regulates the adhesion and motility of myeloid cells [39], which may influence inflammatory responses in CLD. Given the complexity of Wnt signaling in CLD, precision-targeted therapeutic strategies should consider: (i) cell-specific modulation, involving the development of selective Wnt regulators; (ii) combination therapies, such as integrating Wnt modulation with FXR agonists to maintain BA homeostasis; and (iii) timing of intervention, where Wnt activation may be beneficial in acute phases to promote repair, whereas inhibition may be more effective in chronic stages to prevent fibrosis progression. These insights pave the way for refined therapeutic approaches to CLD, balancing the regenerative and pathological aspects of Wnt signaling.

Although the 48 h ANIT model is a well-established acute cholestasis model, CLD in patients typically progresses over a longer duration. Our research primarily focuses on the early prevention of cholestatic injury rather than its long-term progression. Therefore, it is essential to validate our findings using long-term and chronic cholestasis models to assess the broader therapeutic potential of TFNAs. Future studies should explore: (i) whether TFNAs can be developed as an effective treatment for chronic CLD; (ii) the optimal stage of disease progression for TFNAs administration; and (iii) the potential efficacy of TFNAs in combination with other therapeutic agents, such as FXR agonists, to enhance treatment outcomes.

## Conclusion

Our study demonstrates that TFNAs can significantly ameliorate cholestasis by activating the Wnt/β-catenin signaling pathway and promoting ERK1/2 phosphorylation. This provides new insights into the roles of these pathways in CLD progression and highlights the potential of TFNAs as therapeutic agents. Given their antioxidative properties, TFNAs represent a promising strategy for treating CLD in the future.

## Supplementary Material

rbaf017_Supplementary_Data

## Data Availability

The data supporting the findings of this study are available from the corresponding author upon reasonable request.
