# Supplementary material for: Tetrahedral framework nucleic acids ameliorate cholestatic liver disease by activating Wnt/β-catenin signaling and promoting ERK1/2 phosphorylation
Source: Regen Biomater. 2025 Mar 20;12:rbaf017. doi: 10.1093/rb/rbaf017 (PMC12083862; doi:10.1093/rb/rbaf017)

**Supporting Information**

**Tetrahedral Framework Nucleic Acids Ameliorate Cholestatic Liver Disease by Activating Wnt/β-catenin Signaling and Promoting ERK1/2 Phosphorylation**

Jiaming Zhou^†^, Chenxi Tang^†^, Xin Song, Yating Wang, Bingru Lin, Mengchi Lin, Zixin Xu, Shihua Lin, Chengfu Xu^*^, Chaohui Yu^*^

Department of Gastroenterology, Zhejiang Provincial Clinical Research Center for Digestive Diseases, the First Affiliated Hospital, Zhejiang University School of Medicine, Hangzhou, China

**Table S1. The DNA sequence of TFNAs**

| **ssDNAs** | **Sequence (5’-3’)** |
| --- | --- |
| S1 | ATTTATCACCCGCCATAGTAGACGTATCACCAGGCAGTTGAGACGAACATTCCTAAGTCTGAA |
| S2 | ACATGCGAGGGTCCAATACCGACGATTACAGCTTGCTACACGATTCAGACTTAGGAATGTTCG |
| S3 | ACTACTATGGCGGGTGATAAAACGTGTAGCAAGCTGTAATCGACGGGAAGAGCATGCCCATCC |
| S4 | ACGGTATTGGACCCTCGCATGACTCAACTGCCTGGTGATACGAGGATGGGCATGCTCTTCCCG |
| Cy5-S1 | Cy5-ATTTATCACCCGCCATAGTAGACGTATCACCAGGCAGTT GAGACGAACATTCCTAAGTCTGAA |

**Table S2. The primer sequences of target gene**

| **mRNA** | **Product length** | **Primer pairs** | **Sequence (5’-3’)** |
| --- | --- | --- | --- |
| β-Catenin | 281bp | Forward | AAGTTCTTGGCTATTACGACA |
|  |  | Reverse | ACAGCACCTTCAGCACTCT |
| CyclinD1 | 183bp | Forward | GCGTACCCTGACACCAATCTC |
|  |  | Reverse | CTCCTCTTCGCACTTCTGCTC |
| Egfr | 101bp | Forward | GCCATCTGGGCCAAAGATACC |
|  |  | Reverse | GTCTTCGCATGAATAGGCCAAT |
| Erk1 | 248bp | Forward | TCCGCCATGAGAATGTTATAGGC |
|  |  | Reverse | GGTGGTGTTGATAAGCAGATTGG |
| Erk2 | 84bp | Forward | GGTTGTTCCCAAATGCTGACT |
|  |  | Reverse | CAACTTCAATCCTCTTGTGAGGG |
| Gapdh | 123bp | Forward | AGGTCGGTGTGAACGGATTTG |
|  |  | Reverse | TGTAGACCATGTAGTTGAGGTCA |
| Ho-1 | 100bp | Forward | AAGCCGAGAATGCTGAGTTCA |
|  |  | Reverse | GCCGTGTAGATATGGTACAAGGA |
| Jagged1 | 150bp | Forward | CCTCGGGTCAGTTTGAGCTG |
|  |  | Reverse | CCTTGAGGCACACTTTGAAGTA |
| Notch1 | 74bp | Forward | GATGGCCTCAATGGGTACAAG |
|  |  | Reverse | TCGTTGTTGTTGATGTCACAGT |
| Nrf2 | 140bp | Forward | TCTTGGAGTAAGTCGAGAAGTGT |
|  |  | Reverse | GTTGAAACTGAGCGAAAAAGGC |
| Sod2 | 113bp | Forward | CAGACCTGCCTTACGACTATGG |
|  |  | Reverse | CTCGGTGGCGTTGAGATTGTT |
| Yap | 180bp | Forward | TACTGATGCAGGTACTGCGG |
|  |  | Reverse | TCAGGGATCTCAAAGGAGGAC |

**Table S3. The antibodies for western blot**

| **Antibodies** | **Source** | **Catalog** | **Dilution** |
| --- | --- | --- | --- |
| Mouse monoclonal anti-GAPDH | Proteintech | 60004-1-Ig | 1:50,000 |
| Rabbit polyclonal anti-NRF2 | Proteintech | 16396-1-AP | 1:2,000 |
| Rabbit monoclonal anti-β-Catenin | Cell Signaling Technology | 8480 | 1:1,000 |
| Rabbit monoclonal anti-HO-1 | Cell Signaling Technology | 43966 | 1:1,000 |
| Rabbit polyclonal anti-WNT3A | ABclonal | A0642 | 1:1,000 |
| Rabbit monoclonal anti-ERK1/2 | ABclonal | A4782 | 1:1,000 |
| Rabbit monoclonal anti-phospho-ERK1-T202 + ERK2-T185 | ABclonal | AP0485 | 1:1,000 |
| Rabbit monoclonal anti-EGFR | ABclonal | A23381 | 1:1,000 |
| Rabbit monoclonal anti-Vinculin | ABclonal | A2752 | 1:2,000 |
| Rabbit monoclonal anti-SOD2 | ABclonal | A19576 | 1:2,000 |

**Table S4. The antibodies for IHC and IF**

| **Antibodies** | **Source** | **Catalog** | **Dilution** |
| --- | --- | --- | --- |
| Rabbit monoclonal anti-β-Catenin | ABclonal | A19657 | 1:100 |
| Rabbit polyclonal anti-Cytokeratin 19 | Proteintech | 10712-1-AP | 1:3,000 |
| Mouse monoclonal anti- Myeloperoxidase | Servicebio | GB15224 | 1:800 |
| Rabbit monoclonal anti-F4/80 | Cell Signaling Technology | 70076 | 1:400 |
| Rabbit monoclonal anti-Ki-67 | Cell Signaling Technology | 12202 | 1:200 |
| Rabbit monoclonal anti-Ly6g | Abcam | ab238132 | 1:800 |
| Goat Anti-Rabbit IgG H&L (Alexa Fluor® 488) | Abcam | ab150077 | 1:500 |
| Goat Anti-Mouse IgG H&L (Alexa Fluor® 594) | Abcam | ab150116 | 1:500 |
| Anti-Rabbit IgG (HRP-Ploymer) | Biosharp | BL730A-3 | / |

**Figure S1.** (A) Stability of TFNAs in FBS. (B) Biodistribution of TFNAs in mice. (C) Biocompatibility assessment of TFNAs in mice. Abbreviation: FBS, fetal bovine serum; TFNAs, tetrahedral framework nucleic acids.

**
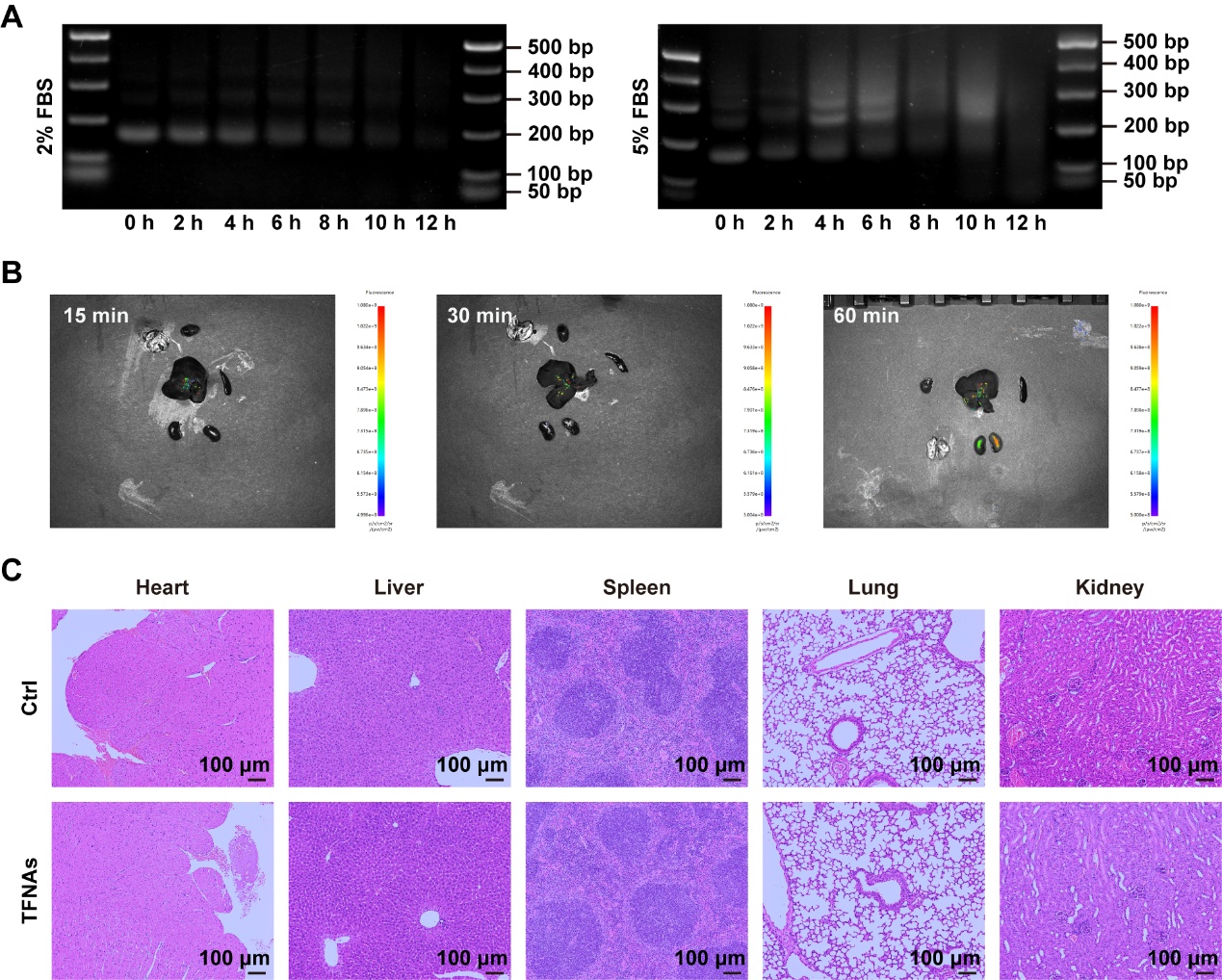
**

**Figure S2.** (A) Stability of TFNAs in cells after ANIT treatment, analyzed by confocal microscopy (*red*: TFNAs; *green*: cytoskeleton; *blue*: nuclei). (B-E) Calcein AM/PI live/dead viability assay of AML12 cells (*n* = 4). The data are presented as the mean ± SD. **P* < 0.05, ***P* < 0.01, ****P* < 0.001, *****P* < 0.0001 vs. the negative control group. ^#^*P* < 0.05, ^##^*P* < 0.01, ^###^*P* < 0.001, ^####^*P* < 0.0001 vs. the ANIT-treated control group. Abbreviation: ANIT, α-naphthyl isothiocyanate; Calcein, calcein acetoxymethyl ester; PI, propidium iodide; TFNAs, tetrahedral framework nucleic acids.


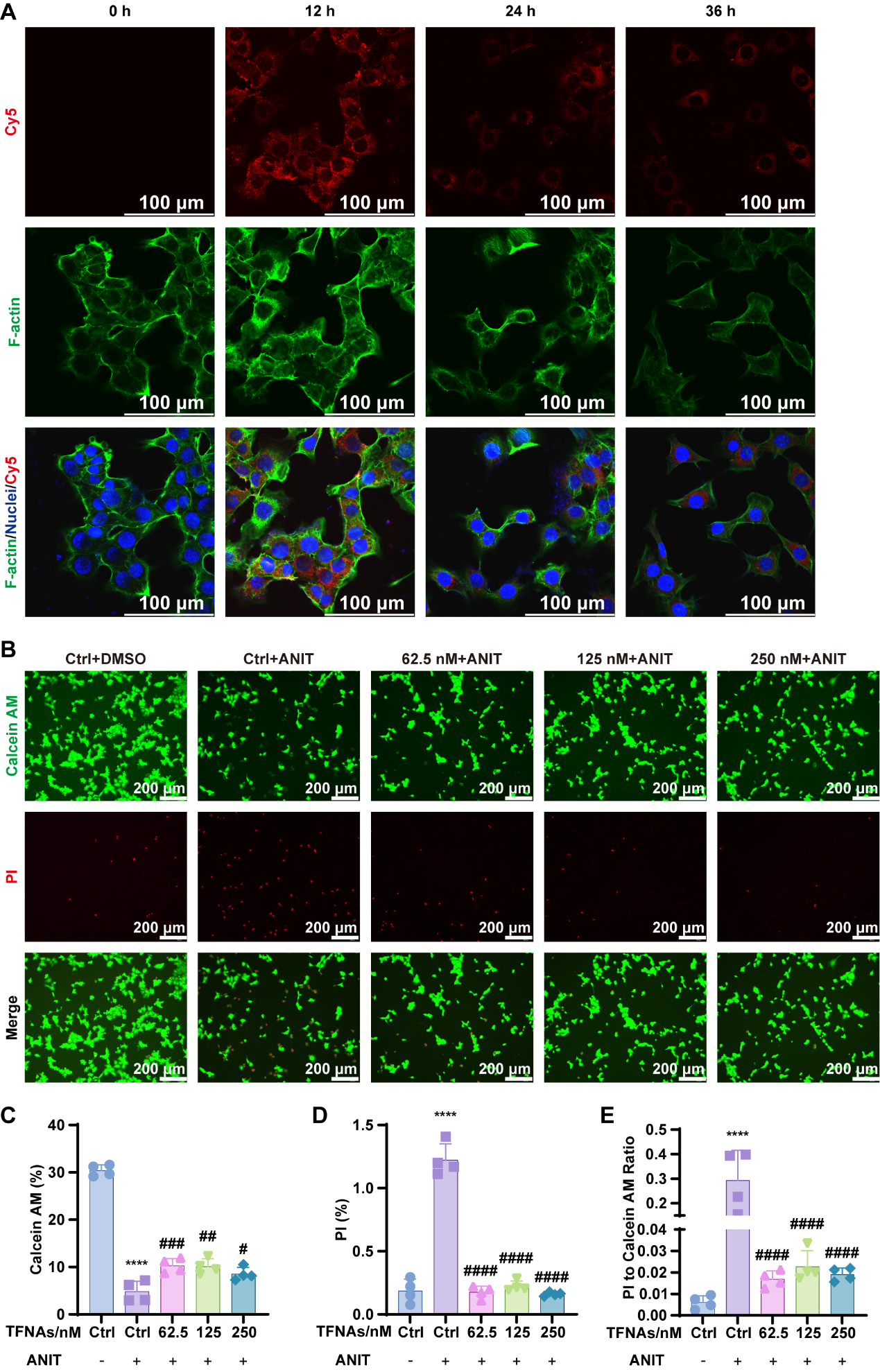


**Figure S3.** (A) IHC staining of F4/80 in liver sections (*n* = 5). (B) Quantitative evaluation of F4/80-positive area (*n* = 5). (C) Immunofluorescence analysis of MPO and F4/80 (*red*: MPO; *Green*: F4/80; *Blue*: nuclei) (*n* = 5). The data are presented as the mean ± SD. **P* < 0.05. Abbreviation: ANIT, α-naphthyl isothiocyanate; MPO, myeloperoxidase; TFNAs, tetrahedral framework nucleic acids.


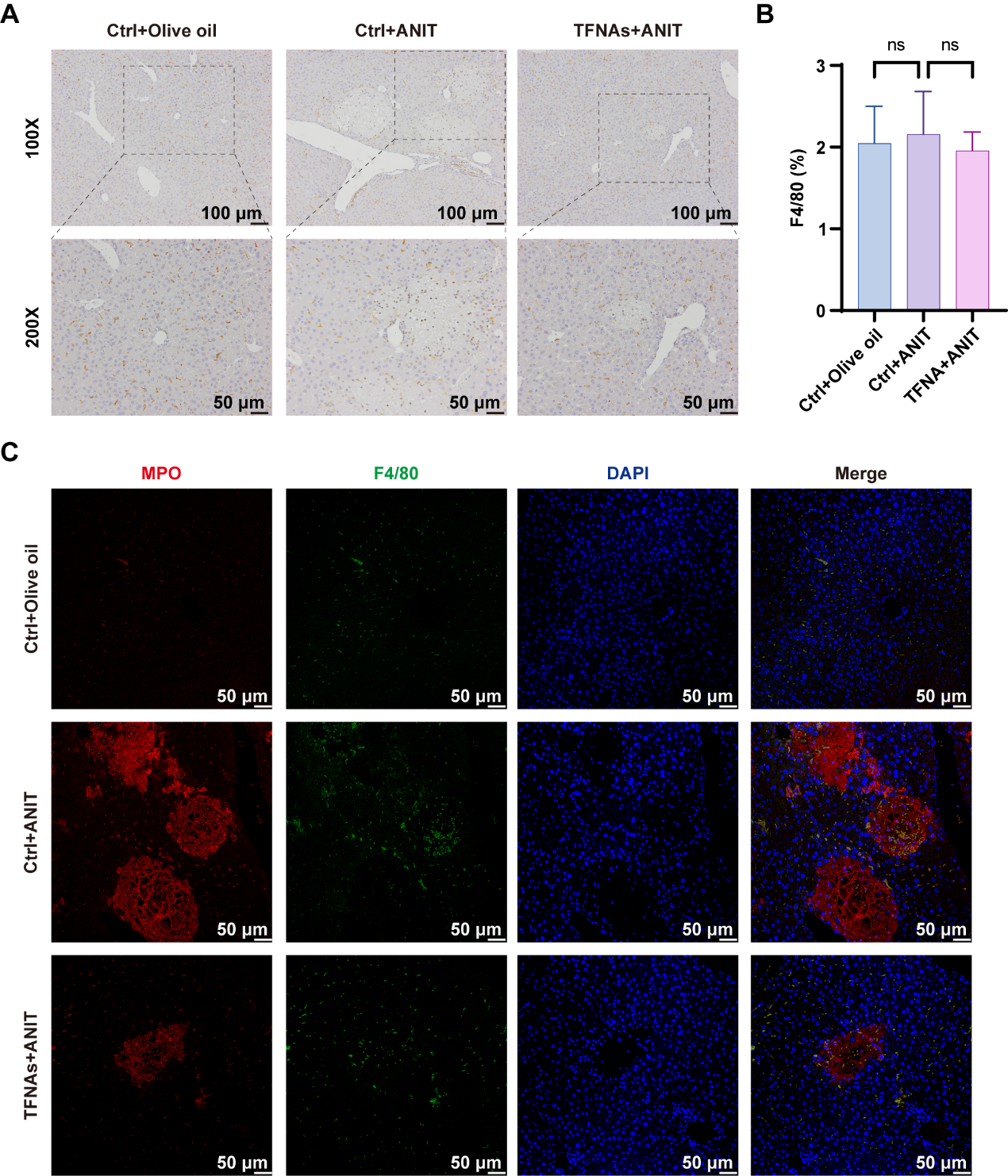

Supplement: rbaf017_Supplementary_Data [file rbaf017_supplementary_data.zip › Supporting Information.docx]
